# The Critical Impact of HIF-1α on Gastric Cancer Biology

**DOI:** 10.3390/cancers5010015

**Published:** 2013-01-10

**Authors:** Yoshihiko Kitajima, Kohji Miyazaki

**Affiliations:** 1 Department of Surgery, Saga University Faculty of Medicine, Saga 849-8501, Japan; 2 Department of Surgery, NHO Higashisaga Hospital, Saga 849-0101, Japan; 3 Saga University Faculty of Medicine, Saga 849-8501, Japan

**Keywords:** HIF-1α, hypoxia, gastric cancer

## Abstract

Hypoxia inducible factor-1 (HIF-1) monitors the cellular response to the oxygen levels in solid tumors. Under hypoxia conditions, HIF-1α protein is stabilized and forms a heterodimer with the HIF-1β subunit. The HIF-1 complex activates the transcription of numerous target genes in order to adapt the hypoxic environment in human cancer cells. In gastric cancer patients, HIF-1α activation following extended hypoxia strongly correlates with an aggressive tumor phenotype and a poor prognosis. HIF-1α activation has been also reported to occur via hypoxia-independent mechanisms such as PI3K/AKT/mTOR signaling and ROS production. This article argues for the critical roles of HIF-1α in glucose metabolism, carcinogenesis, angiogenesis, invasion, metastasis, cell survival and chemoresistance, focusing on gastric cancer.

## 1. Introduction

Gastric cancer is one of the leading causes of cancer death in East Asia, including Japan. Like other solid tumors, gastric cancers involve extensive areas of hypoxia. Recently, increasing evidence has demonstrated that hypoxia is an important micro-environmental factor in promoting tumor progression.

**Figure 1:**
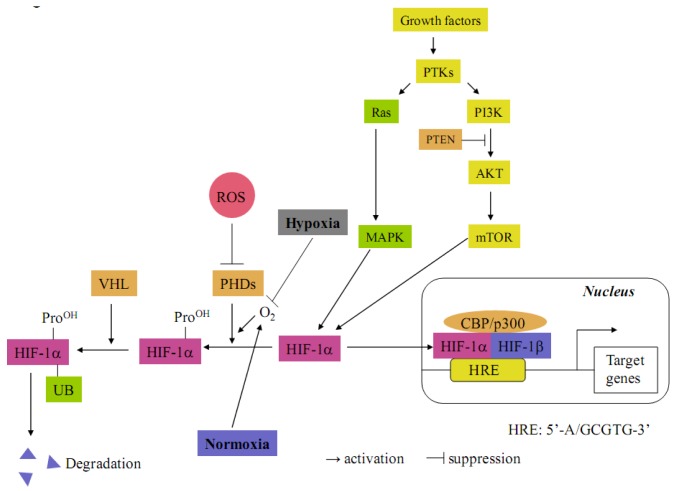
Molecular mechanisms of HIF-1α activation. Under normoxia, HIF-1α is hydroxylated on proline residues by prolyl hydroxylase domain proteins (PHDs). Prolyl-hydroxylated HIF-1α is bound by the von Hippel-Lindau tumor suppressor protein (VHL), which recruits an E3-ubiquitin ligase that targets HIF-1α for proteasomal degradation. Under hypoxia, the prolyl hydroxylation reaction on HIF-1α is inhibited by O_2_ deprivation. HIF-1α degradation is inhibited and translocated into the nucleus where it couples with HIF-1β. The heterodimer then binds together with CBP/p300 to hypoxia-response element (HRE) in the promoters of target genes. HIF-1α proteins are also stabilized by intracellular reactive oxygen species (ROS), which block PHD activities. HIF-1α can also be regulated by oxygen-independent mechanisms. It can be activated by mutations of PTEN and VHL. In addition, HIF-1α can be activated by hyperactivity of the RAS/MAPK and PI3K-AKT-mTOR pathways following growth factor protein tyrosine kinase (PTK) signaling.

The transcription factor hypoxia-inducible factor 1 (HIF-1) constitutes the principal mediator of cellular adaptation to hypoxia. HIF-1 is a heterodimeric protein consisting of a constitutively expressed β-subunit (HIF-1β), known as the aryl hydrocarbon receptor nuclear translocator and hypoxia-inducible α (HIF-1α) subunit [1,2] (Figure 1). Under normoxia conditions, prolyl hydroxylases (PHDs) hydroxylate the cite-specific proline residues of HIF-1α in a reaction that uses O_2_ as a substrate. The modified HIF-1α interacts with von Hippel-Lindau (VHL), which is part of the E3 ubiquitin ligase complex targeting HIF-1α for 26S proteasomal degradation. Under hypoxia, HIF-1α is stabilized due to the lack of O_2_ and dimerizes with HIF-1β interacting with the co-activator CBP/p300 to bind to the hypoxia response element (HRE, 5'-G/ACGTG-3') on the promoter region in various target genes [1,2]. HIF-1α can also be regulated through oxygen-independent mechanisms in a cell type-specific manner (Figure 1) [3,4]. For instance, it can be activated by oncogenic mutations of PTEN, VHL, the RAS/MAPK pathway and the PI3K-Akt-mTOR pathway. Furthermore, HIF-1α is also stabilized by reactive oxygen species (ROS), which block PHD activities [5]. To date, HIF-1 is known to transcriptionally upregulate more than 100 genes [6,7].

HIF-1α overexpression is associated with increased mortality in patients with various tumors, including gastric cancers [5,8]. This association is primarily based on the HIF-1-mediated regulation of genes that play pivotal roles in the central features of cancer pathogenesis such as angiogenesis, invasion, metastasis and anti-apoptosis.

This review briefly summarizes the crucial roles that HIF-1α plays in the glucose metabolism, carcinogenesis, angiogenesis, invasion, metastasis, anoikis and chemoresistance in gastric cancer.

## 2. Glucose Metabolism

**Figure 2:**
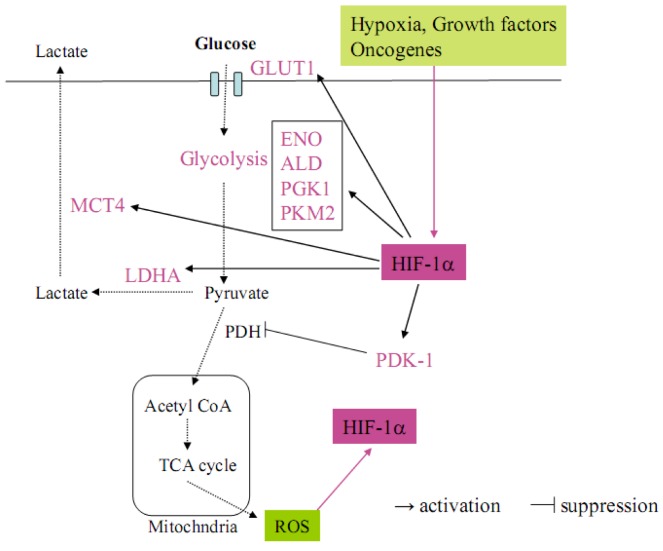
The Warburg effect in cancer cells, in which HIF-1α regulates the target genes related to glucose metabolism. HIF-1α is activated by several factors such as hypoxia, growth factors, oncogenes and intracellular ROS (pink arrow). Activated HIF-1α upregulates glucose transporter GLUT1 to increase the intake of glucose into cancer cells. HIF-1α accelerates the glycolysis step by upregulating ENO, ALD, PGK1 and PKM2 and metabolizing pyruvate. Pyruvate is not converted to acetyl CoA because HIF-1α upregulates PDK-1, which inhibits PDH. On the other hand, pyruvate is metabolized to lactate and transported out of cancer cells by the HIF-1α target genes LDHA and MCT4.

Metabolic abnormalities are one of the critical hallmarks of cancer [9]. As early as the 1920s, Otto Warburg observed that cancer cells generally use glycolysis rather than oxidative phosphorylation for energy [10]. The shift from oxidative to glycolytic metabolism, which is observed in cancer cells, is known historically as the Warburg effect. This metabolic switch allows for the maintenance of redox homeostasis and cell survival in cancer cells. Recently, HIF1α has been revealed to play a central role in regulating the metabolic switch in cancer [9] (Figure 2). In the Warburg effect process, HIF-1α first activates the transcription of SLC2A1 encoding the glucose transporter GLUT1 and increases glucose uptake into cells. Glucose is then metabolized to pyruvate by the actions of glycolytic enzymes such as enolase (ENO) and aldolase (ALD), which are target genes for HIF-1α [9]. Under aerobic conditions, pyruvate is converted to acetyl coenzyme A (AcCoA) by pyruvate dehydrogenase (PDH) for entry into the tricarboxylic acid (TCA) cycle. The TCA cycle along with the electron transport chain (ETC) increases ROS production in mitochondria and further contributes to HIF-1α stabilization (Figure 2) [9]. Under hypoxia, pyruvate is conversely shunted away from mitochondria, whereby HIF-1α upregulates the expression of pyruvate dehydrogenase kinase 1 (PDK1) to inhibit the PDH activity. Moreover, pyruvate is converted to lactate by lactate dehydrogenase A (LDHA) and transported out of cells by monocarboxylate transporter 4 (MCT4). LDHA as well as MCT4 are also targets for HIF-1α (Figure 2) [9]. This is a critical adaptive response to hypoxia because, in HIF-1α-deficient mouse embryo fibroblasts (MEFs), the PDK1 expression is not induced by hypoxia and the cells die due to excess ROS production [11]. These reports strongly suggest that HIF-1α regulates the Warburg effect to avoid a lethal production of mitochondrial ROS in cancer cells, which are exposed in hypoxic environments.

Song *et al*. reported that in gastric cancer, HIF-1α, but not HIF-2α, enhances the expressions of glucose metabolism-related genes such as ENO1, pyruvate kinase 2 (PKM2), phosphoglycerate kinase 1 (PGK1), GLUT1 and LDHA in response to hypoxia [12]. This metabolic switch commonly occurs in all kinds of cancers. Therefore, HIF-1α is a fundamental factor regulating cancer-specific glucose metabolism.

## 3. Carcinogenesis

Few reports have thus far studied the carcinogenic role of HIF-1α in gastric cancer. Griffiths *et al*. previously investigated the HIF-1α expression during carcinogenesis in *Helicobacter pylori* (*H. pylori*) infection [13]. In this study, the HIF-1α expression was immunohistochemically analyzed using normal mucosa and tissues affected by *H. pylori*-associated gastritis, intestinal metaplasia, dysplasia and intestinal adenocarcinoma. No expression of HIF-1α was observed in the normal gastric mucosa, while the expression increased in density and intensity with sequential progression from *H. pylori* infected mucosa to carcinoma. The author summarized that nonhypoxic stabilization of HIF-1α by ROS produced from *H. pylori* plays a role in the stimulation of cell proliferation and protection from apoptosis. In addition, HIF-1α induces genetic instability and provides molecular insights into the mechanisms underlying hypoxia-induced genetic instability [14]. This *H. pylori*-induced HIF-1α activation might lead to the progression of carcinogenesis in gastric mucosa.

## 4. Angiogenesis

Angiogenesis is essential for tumor growth and metastasis. Vascular endothelial growth factor (VEGF) is one of the major factors that contribute to angiogenesis and metastasis in numerous tumor types, and VEGF overexpression is associated with tumor progression and poor clinical outcomes [15,16,17,18]. HIF-1α is known to transcriptionally upregulate the VEGF expression in gastric cancer [19]. Stoeltzing *et al*. previously demonstrated using dominant-negative plasmids of HIF-1α that suppression of HIF-1α decreases VEGF secretion in transfected gastric cancer cells and impairs gastric tumor growth, angiogenesis and vessel maturation in a nude mice model [19]. In that study, the author suggested that HIF-1α not only regulates the VEGF expression in cancer cells, but also contributes to the formation of a complex proangiogenic microenvironment in gastric tumors, thereby affecting the vessel morphology and function. Lang *et al*. revealed another pathway of HIF-1α-mediated angiogenesis in gastric cancer [20]. They showed that the mammalian target of rapamycin (mTOR) is highly activated in gastric cancer specimens. In their study, rapamycin treatment effectively blocked the HIF-1α activation in a gastric cancer cell line under hypoxia and reduced the size of the CD-31-positive vessel area, leading to decreased tumor growth in a subcutaneous implantation model. These results suggest that PI-3K/AKT/mTOR blockade leads to a reduced HIF-1α expression and inhibits tumor angiogenesis in gastric cancer [20].

## 5. Cancer Invasion and Metastasis

Invasion, followed by metastasis to distant organs, is a major problem that needs to be solved in order to achieve a complete cure for cancer. The hypoxia/HIF-1α pathway is a central player that accelerates motility, invasion and metastasis in solid cancers that harbor a hypoxic environment inside. To date, numerous studies have addressed the molecular mechanisms underlying invasion/metastasis related to hypoxia/HIF-1α in human cancers [5]. Our group has previously studied the invasion mechanisms derived from hypoxia/HIF-1α in hepatocellular carcinomas and pancreatic cancer in which MMPs and HGF/HGFA/c-Met activation occur downstream of the hypoxia/HIF-1α axis and act to increase cancer invasion [21,22,23]. In gastric cancer, various mechanisms of invasion and metastasis activated by hypoxia/HIF-1α have been reported. Kato *et al*. investigated the abilities of cancer invasion and metastasis in hypoxia-resistant scirrhous gastric cancer cells [24]. The authors demonstrated that several markers related to epithelial mesenchymal transition (EMT) such as Snail, Twist, ZEB and Vimentin are upregulated in hypoxia-resistant cells compared with that observed in parental cells. Indeed, the resistant cells demonstrated increased invasion abilities *in vitro* and exhibited morphological changes to a spindle-shape compared with the parental cells [24]. Furthermore, the authors showed that the hypoxia-resistant cells, but not the parental cells, developed multiple metastases to the peritoneum and lymph nodes in mice [24]. These results indicate that a hypoxic environment contributes to EMT, leading to increased metastasis in gastric cancer cells. Rohwer *et al*. established a lentiviral-mediated RNA-interference system to knockdown HIF-1α *in vitro* and explore the functional role of HIF-1α in the metastatic capacity of gastric cancer cells under hypoxia [25]. Consequently, HIF-1α deficient cells showed significant reductions not only in migration and invasion, but also in adhesion to vascular endothelial cells [25]. These *in vitro* data suggest the possibility that hypoxia-induced HIF-1α plays an important role in the systemic spread of cancer cells via accelerating intravasation into vessels. Furthermore, the HIF-1α inhibitor 2-methoxy estradiol (2-ME) significantly reduced *in vitro* metastatic properties, suggesting the possibility for drug treatment to reduce cancer invasion and metastasis [25].

Several studies have identified HIF-1α target genes related to cancer invasion/metastasis in gastric cancer. S100A4 is known as a member of the S100 family of calcium-binding proteins involved in the invasiveness and metastasis of tumors [26]. The expression of S100A4 is significantly increased in several tumors, including gastric cancers [26,27,28]. Zhang *et al*. provided evidence that the S100A4 expression is increased under hypoxia and transcriptionally regulated by HIF-1α in a gastric cancer cell line [28]. These reports demonstrate that the invasion activator S100A4 is directly regulated by the hypoxia/HIF-1α axis in gastric cancer. 67-kDa laminin receptor (67LR) is also known to be implicated in cancer metastasis. Liu *et al*. previously reported that 37LRP, the 67LR precursor, is an HIF-1-dependent hypoxia-induced gene [29]. They showed that 67LR promotes the invasive and *in vivo* metastatic ability of gastric cancer cells via increasing a serine protease family member, Urokinase-type plasminogen activator (uPA) and Matrix metalloproteinase 9 (MMP9) expression. Furthermore, both ERK1/2 and c-Jun NH_2_-terminal kinase (JNK) inhibitors significantly inhibit the hypoxia-induced expression of 67LR and subsequently decrease the uPA and MMP9 expression. Taken together, these results demonstrate that ERK1/2 and JNK kinase regulate the HIF-dependent, hypoxia-induced 67LR expression [29].

On the other hand, another study reported that cancer invasion is accelerated by HIF-1α in a hypoxia-independent fashion. Cysteine-rich 61 (Cyr61/CCN1), one of the members of the CCN family, is implicated in cancer invasion of human malignancies. Lin *et al*. demonstrated that Cyr61-activated PI3K/AKT/mTOR and ERK1/2 signaling both act on HIF-1α protein accumulation [30]. That study finally isolated plasminogen activator inhibitor-1 (PAI-1) as the HIF-1α target that increases the invasive abilities of cancer cells. These results prove that HIF-1α activated by growth factor/signal transduction, but not hypoxia, increases gastric cancer invasion.

## 6. Anoikis Resistance

The ability of transformed epithelial cells to initiate the metastatic cascade relies on their ability to escape anoikis, a default program of apoptosis induction following the loss of integrin anchoring to the extracellular matrix. Rohwer *et al*. previously investigated the function of HIF-1α in anoikis resistance and anchorage-independent growth in gastric cancer [31]. They showed that inhibition of HIF-1α via RNA interference results in the upregulation of α5 integrin on the cell surface of gastric cancer cells, whereas other integrins remain unaffected. HIF-1α deficient gastric cancer cells display significantly increased anoikis susceptibility due to an upregulated α5 integrin expression, suggesting a pivotal role for HIF-1α in anoikis control via suppression of α5 integrin [31]. The suppressive effects of HIF-1α on α5 integrin might allow gastric cancer cells to survive in vessels or the peritoneal cavity, resulting in distant metastasis and peritoneal dissemination.

## 7. Chemoresistance

**Figure 3:**
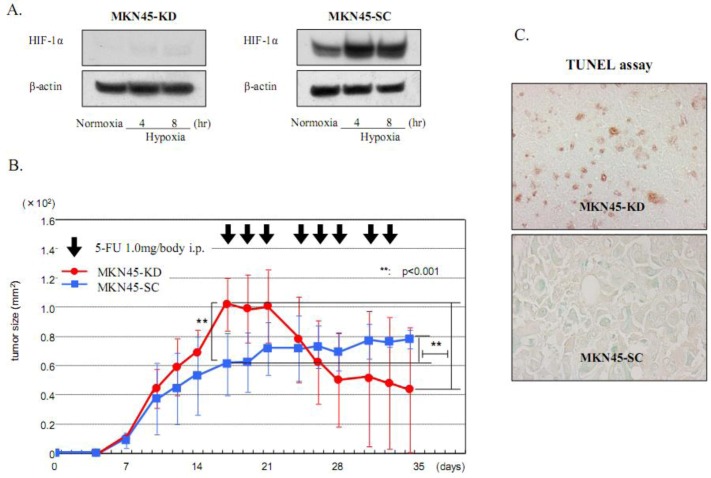
A deficient HIF-1α expression increases chemosensitivity to 5-FU in the gastric cancer cells MKN-45. (**A**) A Western blot analysis of HIF-1α in the HIF-1α-deficient gastric cancer cell line MKN45-KD in which HIF-1α siRNA plasmids were transfected. Hypoxic induction of HIF-1α was completely knocked down in MKN45-KD compared with that observed in the control cell line MKN45-SC. (**B**) Effects of HIF-1α knockdown on sensitivity in xenografts of nude mice. 5-FU treatment was administered three times a week starting on Day 17. The tumor size before treatment was significantly larger in the MKN45-KD tumors than in the MKN45-SC tumors. The response rate to 5-FU, which was estimated based on tumor size on Day 35, was significantly better in the KD tumors than in the SC tumors (41.7% ± 38.3% *vs*. 141.38 ± 48.1%). (**C**) A TUNEL assay showed a significant increase in the number of apoptotic cells with nuclear staining in the HIF-1α KD tumors after 5-FU treatment.

**Figure 4:**
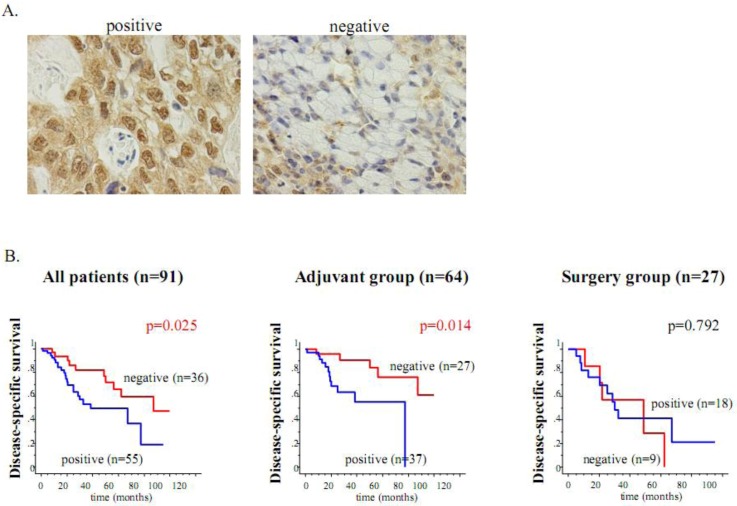
The HIF-1α expression is an unfavorable determinant of relapse in gastric cancer patients who undergo adjuvant 5-FU chemotherapy. (**A**) Immunohistochemical staining of HIF-1α in gastric cancer specimens. Nuclear staining of the HIF-1α expression is shown in the left panel, while the expression is negatively observed in the right panel. (**B**) Kaplan-Meier estimates of disease-specific survival (DSS) of the patients according to the HIF-1α expression. Among all 91 patients, the HIF-1α-negative cases showed significantly longer DSS than the HIF-1α-positive cases. Among the 64 patients who underwent adjuvant chemotherapy after surgery, the DSS of the HIF-1α-negative cases was also significantly longer than that of the HIF-1α-positive cases. On the other hand, in the 27 patients in the surgery alone group, the HIF-1α expression did not contribute to patient survival.

Previous studies have reported that HIF-1α is an important contributor involved in drug resistance against cancer chemotherapy [1,2,8]. Our group previously established two gastric cancer cell lines, MKN45-KD and MKN74-KD, with a deficient HIF-1α expression using siRNA transfection [32]. We elucidated that HIF-1α knockdown cells are more sensitive to 5-FU than the control cells, MKN45-SC and MKN74-SC, *in vitro*. Furthermore, the effects of HIF-1α knockdown on 5-FU-induced apoptosis are distinctly observed in xenograft tumors, which harbor a hypoxic region inside (Figure 3). Finally, an immunohistochemical analysis using 95 resected cancer tissues revealed that the HIF-1α expression is an independent risk factor for relapse in gastric cancer patients who postoperatively undergo 5-FU chemotherapy (Figure 4). In this study, we clearly showed that the HIF-1α expression acts on drug resistance against 5-FU and can be clinically used as a biomarker to predict chemosensitivity to this treatment. Recently, Rohwer *et al*. also addressed the mechanisms underlying the HIF-1α-mediated chemoresistance against 5-FU in gastric cancer [33]. Using siRNA technology, the authors proved that a loss of HIF-1α abrogates, in a p53-dependent manner, the expression of anti-apoptotic NF-κB target genes such as cIAP1 [33]. These two reports strongly indicate that the treatment using siRNA against HIF-1α might be an effective therapy, via increasing 5FU sensitivity in gastric cancer patients. On the other hand, Liu *et al*. demonstrated that the HIF-1α expression is important for vincristine resistance in gastric cancer cells under hypoxia [34]. In addition, the author revealed that the hypoxia-induced HIF-1α expression increases the expression of anti-apoptotic factor Bcl-2 and the multidrug resistant gene MRP1, suggesting a novel mechanism of hypoxia-induced chemoresistance in gastric cancer [34]. The same author recently reported that the 37-kDa laminin receptor precursor (MGr1-Ag/37LRP) is an HIF-1α target gene and exhibits a resistant function against multidrugs, including 5-FU, adriamycin (ADR), cisplatin (CDDP), vincristine (VCR) and etoposide (VP-16), under hypoxia [35]. That study finally demonstrated that ROS production under hypoxia activates MEK signaling, and the ROS-elicited MEK signaling transduces HIF-1α activation, which finally induces the MGr1-Ag/37LRP expression involved in chemoresistance [35].

In addition to chemoresistance, tumor hypoxia is also a known cause of failure to radiotherapy in solid tumors. HIF-1α has been reported to regulate multiple aspects of response to radiation therapy [36]. Recently, Oommen *et al.* revealed that the benzylidene lactam compound KNK437 abrogates hypoxia-induced radioresistance via inhibiting AKT-HIF-1α signaling in human breast cancer and glioma cells [37]. Yu *et al*. demonstrated that TSA acts as a powerful radiosensitizer in Hela cells under hypoxic conditions by down-regulated expression of HIF-1α and VEGF proteins [38]. These reports indicate that HIF-1α acts on radioresistance under hypoxia and the HIF-1α inhibitors might be useful in sensitizing the solid cancers to radiation therapy. At present, few reports have argued for the implication of HIF-1α in radioresistance using gastric cancer cells. In the future, the important role of HIF-1α in radioresistance might be revealed in gastric cancer.

## 8. Conclusions

**Figure 5:**
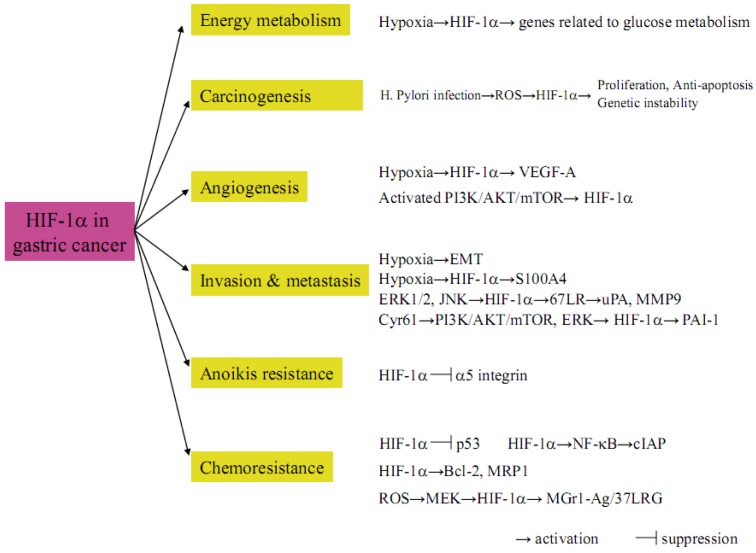
The critical impact of HIF-1α on gastric cancer biology.

Figure 5 summarizes the HIF-1α mediated pathways in gastric cancer biology that are described in this review. These studies suggest the possible rationale that hypoxia-dependent or -independent activation of HIF-1α is a master regulator that accelerates malignant behaviors in gastric cancer. In the future, some drug therapies targeting HIF-1α itself or HIF-1α mediated cascades such as glucose metabolism, carcinogenesis, angiogenesis, invasion, metastasis, apoptosis and chemoresistance might be designed, thereby improving unfavorable outcomes in gastric cancer patients.
